# An update on noninvasive neuromodulation in the treatment of patients with prolonged disorders of consciousness

**DOI:** 10.1111/cns.14757

**Published:** 2024-05-15

**Authors:** Xiaoping Wan, Ye Zhang, Yanhua Li, Weiqun Song

**Affiliations:** ^1^ Department of Rehabilitation Medicine, Xuan Wu Hospital Capital Medical University Beijing China

**Keywords:** neuromodulation, noninvasive interventions, prolonged disorders of consciousness, treatment

## Abstract

**Background:**

With the improvement of emergency techniques, the survival rate of patients with severe brain injury has increased. However, this has also led to an annual increase in the number of patients with prolonged disorders of consciousness (pDoC). Hence, recovery of consciousness is an important part of treatment. With advancing techniques, noninvasive neuromodulation seems a promising intervention. The objective of this review was to summarize the latest techniques and provide the basis for protocols of noninvasive neuromodulations in pDoC.

**Methods:**

This review summarized the advances in noninvasive neuromodulation in the treatment of pDoC in the last 5 years.

**Results:**

Variable techniques of neuromodulation are used in pDoC. Transcranial ultrasonic stimulation (TUS) and transcutaneous auricular vagus nerve stimulation (taVNS) are very new techniques, while transcranial direct current stimulation (tDCS) and transcranial magnetic stimulation (TMS) are still the hotspots in pDoC. Median nerve electrical stimulation (MNS) has received little attention in the last 5 years.

**Conclusions:**

Noninvasive neuromodulation is a valuable and promising technique to treat pDoC. Further studies are needed to determine a unified stimulus protocol to achieve optimal effects as well as safety.

## Graphical abstract

This review delves into the update on noninvasive neuromodulations in the treatment of patients with prolonged disorders of consciousness (pDoC). It introduced the progress made by tDCS, TMS, TUS, MNS, and taVNS, respectively, in pDoC. The comparison was also made with each other. The results could provide new ideas for future research and clinical practice.

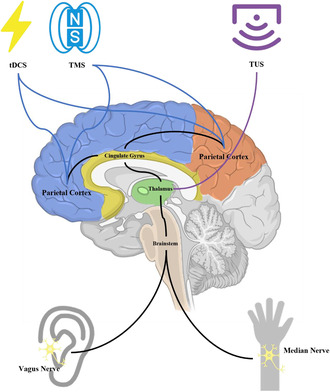

## 1 INTRODUCTION

Prolonged disorders of consciousness (pDoC) are a category of DoC wherein the process of loss of consciousness lasts >28 days after different types of severe brain injuries.^1^ The pDoC can be classified into unresponsive wakefulness syndrome (UWS), which was known as vegetative state (VS) in the past, and minimally conscious state (MCS). In the United States, it is estimated that there were a sum of patients in persistent VS state, ranging from 10,000 to 25,000 in adults and 4000 to 10,000 in children.^2^ As for MCS, there are 280,000 adults and 112,000 children.^3^ A previous review demonstrated that the prevalence in four European countries and Japan varied from 0.2 to 3.4 cases per 100,000 inhabitants in VS and 1.5 cases per 100,000 in MCS.^4^ In China, domestic studies showed that the total number of pDoC is over 500,000, with an increase of 70,000–100,000 patients every year.^5^ The causes of pDoC are more relevant to traumatic brain injury, stroke, and cardiac arrest with attempted cardiac pulmonary resuscitation (CPR). Patients with pDoC may be bedridden for a long time causing several and multiple complications such as hydrocephalus, pulmonary infection, urinary tract infection, and/or paroxysmal sympathetic hyperactivity. These not only create great difficulties for caregivers but also impose economic burdens on both families and society. According to statistics, the annual cumulative medical expenditure for pDoC‐related conditions is 30–50 billion yuan in China.^5^

Hence, identifying an effective intervention for the arousal of consciousness matters. Amantadine is the only recommended pharmacotherapy to hasten functional recovery in traumatic pDoC.^6^ However, this medicine is only suitable for traumatic pDoC,^6^ and it lacks efficacy for pDoC of other etiology. Noninvasive neuromodulation has been considered a valuable and promising method to treat pDoC, particularly when focused on improving the level of consciousness.^7^ Nevertheless, the parameters (frequency, targets, and duration) of stimulus protocol settings vary among studies. Further studies are needed to help to develop a uniform program for noninvasive neuromodulation, in terms of both safety and efficacy for pDoC patients.

Therefore, in this review, we discuss the advances in noninvasive neuromodulation in the treatment of pDoC in the last 5 years and analyze the parameters and efficacy of every technique. We believe this review will provide a basis for optimal stimulation protocol selection for future clinical use.

## 2 UPDATE ON TRANSCRANIAL DIRECT CURRENT STIMULATION

Transcranial direct current stimulation (tDCS) is one of the most representative noninvasive neuromodulation interventions. It regulates brain activity through the electrodes by delivering a weak current; it does not directly induce brain activities. However, it achieves modulation by changing the neuronal membrane potential. The anodic electrode depolarizes the neuronal membrane to achieve excitation, while the cathodic electrode hyperpolarizes it to achieve inhibition.^8^ The underlying mechanism is based on the neuroplasticity theory of long‐term potentiation and long‐term depression.^9^ In the last 5 years, research in tDCS has made progress. The characteristics of these are seen in Table 1.

**TABLE 1:** Studies of tDCS published in the last 5 years.

Literature	Study design	Number of patients and diagnosis	Number of real tDCS sessions	Targets	Evaluation	Behavioral outcomes	tDCS induced effects	Characteristics
Thibaut et al. 2018^18^	Retrospective study	21 MCS	1	Left DLPFC	CRS‐R/EEG (power spectrum/spectral connectivity‐graph theory)	Group level (−)	EEG: Main in responders: Theta activity (+) Theta band connectivity (+)	S‐S‐C tDCS
Bai et al. 2018^17^	Sham controlled, crossover	8 MCS; 9 UWS	1	Left DLPFC	CRS‐R/EEG (coherence)	Group level (−)	EEG: Main in MCS group: Theta band (+) Gamma band (−)	S‐S‐C tDCS
Martens et al. 2018^30^	Double‐blind, crossover	27 MCS	20	Left DLPFC	CRS‐R/AE	Group level (+)	—	M‐S‐HB tDCS
Martens et al. 2019^16^	Double‐blind, crossover	6 MCS; 4 UWS	1	Left or Right M1 (the most effected side)	CRS‐R	Group level (−)	—	S‐S‐C tDCS
Wu et al. 2019^26^	Randomized sham‐controlled	7 MCS; 8 UWS	10	Left DLPFC or Right DLPFC	CRS‐R/EEG (functional connectivity)	Group level (−)	EEG: The excitability of the prefrontal cortex (+)	M‐S‐C tDCS
Cavinato et al. 2019^23^	Double‐blind, crossover	12 MCS; 12 UWS	10	Left DLPFC	CRS‐R/WNSSP/EEG (power spectra and coherence analysis)	Group level (+)	EEG: In MCS group: Alpha power and coherence (+) Beta power and coherence (+) In UWS group: Slow frequencies power (−) Slow frequencies coherence (+)	M‐S‐C tDCS
Zhang et al. 2019^22^	Nonrandomized controlled	5 MCS; 8 UWS	20	Left DLPFC	CRS‐R/ERP/PET	MCS group (+)	ERP: In MCS group: P300 (+) In UWS group: P300 (−)	M‐S‐C tDCS
Li et al. 2019^24^	Double‐blind	80 after TBI	24	Left DLPFC	GCS/GOS/EEG/BEAP/SEP/AE	—	—	M‐S‐C tDCS, study protocol
Cai et al. 2019^27^	Nonrandomized controlled	18 MCS; 10 UWS	14	Precuneus	CRS‐R/resting‐state EEG	24/28 patients (+)	EEG: In responders: Delta band power (−) Alpha band power (+)	M‐S‐HD tDCS
Guo et al. 2019^25^	Nonrandomized controlled	6 MCS; 5 UWS	28	Precuneus	CRS‐R/EEG (functional connectivity)	9/11 patients (+)	EEG: Delta band coherence (−)	M‐S‐HD tDCS
Straudi et al. 2019^29^	Open label pilot, nonrandomized controlled	10 MCS after TBI	10	Bilateral M1	CRS‐R/EEG	MCS group (+)	EEG: Upper alpha (11–13 Hz) power (+)	M‐T‐C tDCS
Lin et al. 2019^28^	Two‐case	1 MCS; 1 control	14	Bilateral inferior parietal lobes	CRS‐R/GCS/FOUR/CNC/ DTF/fMRI (global brain connectivity)	One MCS (+)	EEG: Gamma DTF value (+); fMRI: Normalized activity (+)	M‐T‐C tDCS+TMS
Carrière et al. 2020^14^	Double‐blind, crossover	1 eMCS; 4 MCS+ 6 MCS−	1	Left DLPFC	CRS‐R/EEG (spectral power/ connectivity)	Group level (+)	EEG: Alpha band power (+) Theta band power (+) wSMI connectivity (+) wPLI connectivity (+)	S‐S‐C tDCS
Hermann et al. 2020^13^	Prospective open‐label	4 eMCS; 32 MCS; 24 UWS	1	Left DLPFC	CRS‐R/EEG (spectral power/complexity/functional connectivity/ERP)	20% (12/60) patients (+)	EEG: In responders: Theta‐alpha band power (+) Theta‐alpha band functional connectivity (+) P300 (+)	S‐S‐C tDCS
Mensen et al. 2020^12^	—	4 MCS; 3 UWS	1	Left DLPFC	TMS‐EEG/resting‐state EEG	Group level (+)	TMS‐EEG: Slow activity (−) Resting‐state EEG: The incidence of slow waves (−)	S‐S‐C tDCS
Wang et al. 2020^20^	Nonrandomized controlled	9 MCS; 2 UWS	28	Precuneus	CRS‐R/EEG (MMN)	Group level (+)	EEG: MMN amplitudes (+)	M‐S‐HD tDCS
Zhang et al. 2020^21^	Nonrandomized controlled	20 MCS; 15 UWS	28	Precuneus	CRS‐R/resting‐state EEG (spectral connectivity‐dwPLI/graph theory)	MCS group (+)	EEG: Beta and gamma bands functional brain networks (+)	M‐S‐HD tDCS
Martens et al. 2020^15^	Double‐blind, crossover	6 eMCS; 23 MCS; 17 UWS	1	Bilateral frontoparietal cortex	CRS‐R/resting‐state EEG	Group level (−)	EEG: Low‐frequency bands (1–8 Hz) complexity (−)	S‐M‐C tDCS
Martens et al. 2021^10^	Randomized controlled crossover	16 MCS	1	Bilateral prefrontal cortex	CRS‐R/EEG (real‐time spectral entropy)	—	—	S‐T‐C tDCS, study protocol
Zhang et al. 2021^19^	Open‐label, controlled	48 MCS; 57 UWS	80	Prefrontal+Bilateral fronto‐temporo‐parietal cortex+Left DLPFC	CRS‐R/mGOS/EEG (nonlinear EEG index)	Group level (+)	EEG: C‐ApEn (+)	M‐M‐C tDCS
Barra et al. 2022^11^	Double‐blind sham‐controlled	2 eMCS 4 MCS+ 6 MCS−	1	Left prefrontal cortex	CRS‐R/EEG (functional connectivity)	Group level (+)	EEG: Theta functional connectivity (+)	S‐S‐C tDCS
Zhang et al. 2022^31^	Nonrandomized controlled	31 MCS	20	Left DLPFC	CRS‐R/ERP	54.8% (17/31) MCS (+)	EEG: In MCS responders: P300 (+)	M‐S‐C tDCS
Yu et al. 2022^32^	Randomized sham‐controlled	33 UWS after stroke	10	Left DLPFC	CRS‐R/EEG/PSG /GOS‐E	Group level (+)	PSG Total sleep time (+) Sleep structure changed EEG: Alpha band power (+) Beta band power (+) Gamma band power (+) Functional connectivity (+)	M‐S‐C tDCS

Abbreviations: AE, adverse reaction; BAEP, brainstem auditory‐evoked potentials; C‐ApEn, the cross approximate entropy; CNC, the Coma/Near Coma Scale; CRS‐R, JFK Coma Recovery Scale‐Revised; DLPFC, the dorsolateral prefrontal cortex; DTF, directional transfer function; dwPLI, debiased weighted phase lag index; EEG, electroencephalography; ERP, event‐related potential; fMRI, functional magnetic resonance imaging; FOUR, Full Outline of Unresponsiveness Scale; GCS, Glasgow Coma Scale; GOS, Glasgow Outcome Scale; M1, the primary motor cortex; MCS, minimally conscious state; mGOS, modified Glasgow Outcome Scale; M‐M‐C tDCS, multi session‐multi targets‐conventional tDCS; MMN, mismatch negativity; M‐S‐C tDCS, multisession‐single target‐conventional tDCS; M‐S‐HB tDCS, multisession‐single target‐homebased tDCS; M‐S‐HD tDCS, multisession‐single target‐ high‐definition tDCS; M‐T‐C tDCS, multisession‐two targets‐conventional tDCS; PET, positron emission tomography; PSG, polysomngraphy; SEP, somatosensory‐evoked potential; S‐M‐C tDCS, single session‐multi targets‐conventional tDCS; S‐S‐C tDCS, single session‐single target‐conventional tDCS; S‐T‐C tDCS, single session‐two targets‐conventional tDCS; tDCS, transcranical direct current stimulation; TMS, transcranial magnetic stimulation; TMS‐EEG, transcranial magnetic stimulation combined with electroencephalography; UWS, unresponsive wakefulness state; WNSSP, Western neuro sensory stimulation profile; wPLI, weighted phase lag index; wSWI, weighted symbolic mutual information.

### 2.1 Form of the stimulation

Conventional tDCS delivers a current through pad electrodes, with the intensity typically set at 1–2 mA, and the duration at 20 min. From 2018 to 2022, nine papers reported the transient effects of a single session of tDCS.^10^, ^11^, ^12^, ^13^, ^14^, ^15^, ^16^, ^17^, ^18^ Among these studies, the coma recovery scale‐revised (CRS‐R) was the most commonly used to evaluate the state of consciousness. Electrophysiology contains many aspects, including power spectra, event‐related potential (ERP), complexity, connectivity, and transcranial magnetic stimulation combined with electroencephalography (TMS‐EEG). The methods mentioned above have all been used to evaluate the validity of tDCS on pDoC.

With regard to power spectra, Carrière et al.^14^ found that there was an increase in relative power in the theta band in the frontal, parietal, and occipital regions, while there was an increase in the alpha band in the central regions. Hermann et al.^13^ reported that the responders showed a significant increase in theta power in the parietal cortices. Furthermore, an increase in alpha power was observed. However, two studies^15^, ^17^ did not find any significant results in power for any bandwidths. As for the EEG complexity, a trend of an increase in the theta‐alpha band was observed in the responders.^13^ In the theta and delta bands, tDCS had an impact on the Lemple‐Ziv‐Welch complexity.^15^ In terms of connectivity, Barra et al.^11^ found an increase in the theta frontal connectivity after tDCS. Hermann et al.^13^ observed an increase in functional connectivity in the theta‐alpha band for responders. Another study observed an increase in weighted symbolic mutual information (wSMI) alpha connectivity in the parietal region and an increase in weighted phase lag index (wPLI) alpha connectivity in the fronto‐parietal regions.^14^ Another study found that the responders showed an increased theta band connectivity.^18^ Only one analyzed its influence under TMS‐EEG.^12^ After a single session of tDCS, the overall evoked slow activity was reduced. In Hermann et al's study, patients who were classified as responders of tDCS showed larger and more sustained P300 in terms of the event‐related potential (ERP).^13^

Although few significant behavioral changes were observed after one therapy session, tDCS caused electrophysiological changes. Based on these single‐session findings, studies assumed that multi‐sessions would perhaps have an enhancing effect that ultimately manifested in behavior. Thus, an increasing number of studies were designed to investigate the effects in the form of multiple repeated sessions. In the last 5 years, 14 related papers were published^19^, ^20^, ^21^, ^22^, ^23^, ^24^, ^25^, ^26^, ^27^, ^28^, ^29^, ^30^, ^31^, ^32^ (Table 1). The number of sessions was not consistent, varying from 10 to 80 times, and the results were inconsistent. Among these 14 studies, 11 showed an increase in CRS‐R scores, seven showed significant changes in the group level (five showed a significant increase in the MCS group^21^, ^22^, ^29^, ^30^, ^31^ and two of which contain the maximum number of sessions reported a significant increase both in the MCS and UWS groups^19^, ^20^). These results confirmed the assumption that multi‐sessions did in fact enhance the effects of tDCS.

**FIGURE 1:**
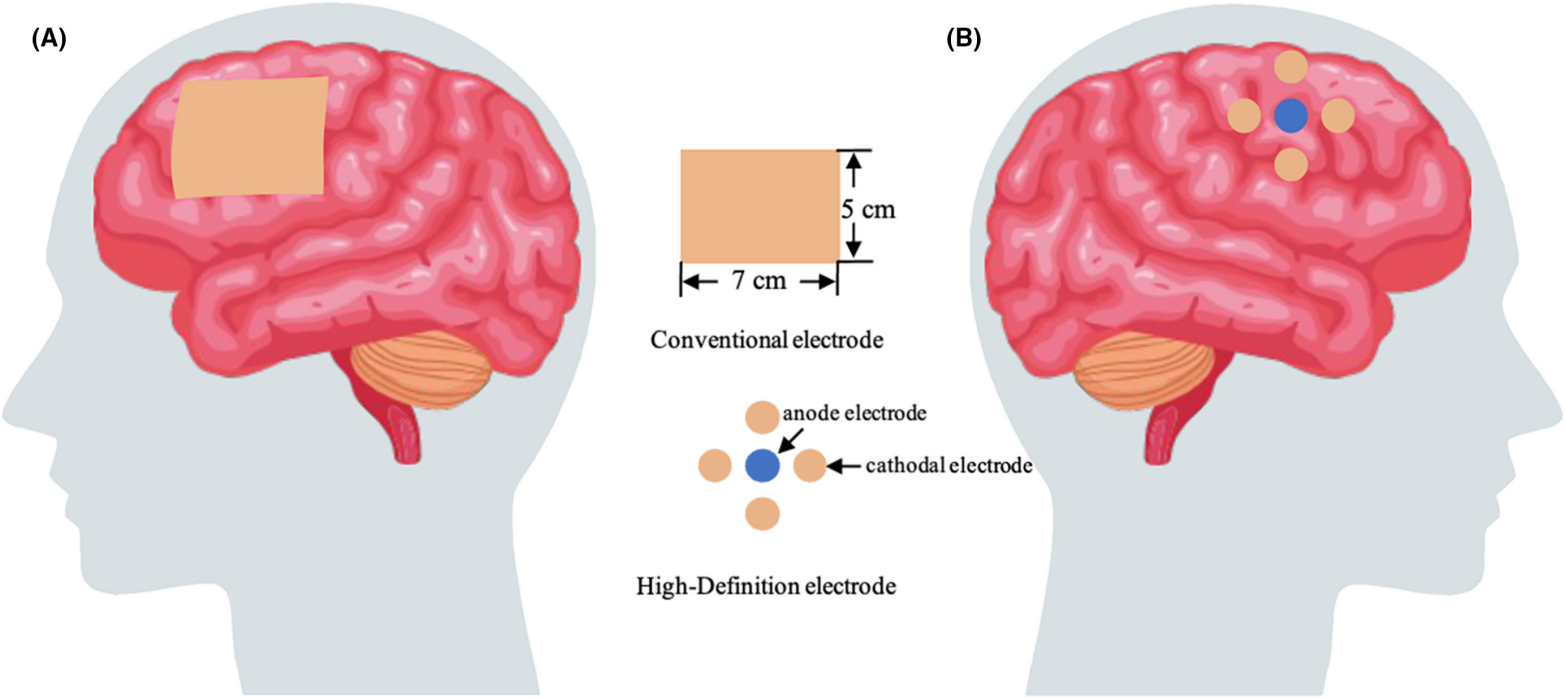
(A) The anode electrode of conventional tDCS. (B) Electrodes of high‐definition tDCS.

Apart from session numbers, tDCS has also made innovations in other formats. Conventional tDCS contains two separate pad electrodes of anode and cathode. The normal size of each electrode is about 5 × 7 cm (Figure 1A). Because of its shape and diffuse brain current flow, it was difficult to localize the real cortical region stimulated by tDCS. To overcome this drawback, researchers have made a modification to conventional tDCS, namely high‐definition tDCS (HD‐tDCS) (Figure 1B), wherein the two large pad electrodes have been discarded, and the new device is equipped with five sets of ring compact scalp electrodes (the diameter of each electrode is <12 mm)—one central anodal electrode and four surrounding cathodal electrodes. Because of its small size, it has the advantages of precise spatial positioning. The HD‐tDCS was reported to have improved multiple aspects of humans' ability that are related to consciousness: motor function, verbal learning, and working memory. It was first applied in pDoC patients in 2019.^27^ Thus far, only four papers^20^, ^21^, ^25^, ^27^ have reported its application in pDoC patients. All of them were about long‐session therapy in 14 days, one was about 14 sessions (once a day),^27^ and three were about 28 sessions (twice a day).^20^, ^21^, ^25^ Twenty‐four of the 28 DoC patients achieved improvement in CRS‐R scores after 14 HD‐tDCS sessions, wherein the responders showed a decrease in delta band and an increase in alpha band in resting‐state EEG.^27^ After 28 sessions of tDCS, pDoC had varying degrees of improvement in CRS‐R scores in the other three studies.^20^, ^21^, ^25^ Apart from the improvement in behavioral scores, electrophysiological studies found that there was a decrease in the delta band of the central‐parietal coherence, an increase in beta and gamma bands of resting‐state functional brain networks, and an improvement in mismatch negative (MMN).

The above‐mentioned studies adequately demonstrated that single stimulation was insufficient for the improvement of consciousness, whereas multi‐sessions fared well. However, multi‐session therapy means long‐term treatment. This may be a limitation for inpatients considering the prolonged hospitalization period. The appearance of home‐based tDCS can well remove this restriction. One study^30^ showed the efficacy and safety of the home‐based tDCS in 2018. After 4 weeks (once a day, 5 days/week) of 20 tDCS sessions, the device showed a moderate effect in DoC patients with no severe adverse events.

Single tDCS treatment has shown its effects on the brain cortex which can be displayed in EEG. However, some researchers found that tDCS alone with TMS might increase the excitability than monotherapy. Yicong Lin et al.^28^ applied tDCS and rTMS combination therapy in a single MCS patient; apart from clinical scores improvement, they also found a significant directional transfer function (DTF) value increase in the gamma band of bilateral posterior regions by EEG analysis. Furthermore, functional magnetic resonance imaging (fMRI) showed that the activities of inferior parietal lobes (IPLs) tended to normalize.

### 2.2 Choice of target

#### 2.2.1 Single

In the protocol of tDCS, target is one of the important parameters. Most studies were initially conducted with single‐target therapy, targeting the dorsolateral prefrontal cortex (DLPFC), primary motor cortex (M1), and precuneus.^11^, ^12^, ^13^, ^14^, ^16^, ^17^, ^18^, ^20^, ^21^, ^22^, ^23^, ^24^, ^25^, ^26^, ^27^, ^30^, ^31^, ^32^ The DLPFC has been a hot spot for tDCS‐stimulated target selection.^12^, ^13^, ^14^, ^17^, ^18^, ^22^, ^23^, ^24^, ^26^, ^30^, ^31^, ^32^ The mechanism underlying the DoC is still unknown. Some researchers believe that the recovery of consciousness is related to the frontoparietal network. The DLPFC is one of the main brain regions of the frontoparietal network and plays an important role in motor control and executive function. Moreover, DLPFC has connections with other cortices, and once activated, it has the ability to transmit excitability to the whole cerebral cortex.

Most of the single‐session tDCS therapy on left DLPFC reported no positive results at the group level, and multi‐sessions showed uniform results. Some of the studies reported significant changes in the MCS group, while others showed no positive results.

Though some positive results have been observed, some researchers thought that DLPFC might not be an ideal target because it is easily damaged in traumatic brain injury (TBI) which is one of the most common causes of pDoC. In this case, investigators make efforts to find new targets. Precuneus is another choice. First, it is one of the components of the frontoparietal network and may play a central role. In the last 5 years, a total of four literature reports were applied to the precuneus.^20^, ^21^, ^25^, ^27^ They all showed that the precuneus was a promising choice to obtain positive results both in clinical scores and electrophysiology examination.

The M1 was another choice though only one study reported its use in tDCS of pDoC. In the evaluation of pDoC, motor function that could be easily observed at the bedside is one of the essential parts. Furthermore, the recovery of motor function may promote the active participation of rehabilitation and accelerate the recovery of consciousness. In the randomized double‐blind sham‐controlled crossover trial,^16^ two patients were observed to have new sign of consciousness, regaining visual pursuit, and object localization, but they had nothing to do with motor function. Similarly, single‐session tDCS in M1 did not significantly improve the total score as well as the score of the motor subscale at the group level.

#### 2.2.2 Multi‐target

The effectiveness induced by tDCS is based on the patients' retention of residual cortical function and relative complete remote neural network. However, pDoC means more severe damage of the neural network. Some researchers believe that because single‐target is not sufficient to activate the entire network of consciousness, multi‐target might be a better approach to achieve activation. Several studies (three studies about two targets^10^, ^28^, ^29^ and two studies about multi‐targets^15^, ^19^) have attempted to use multi‐target therapy for pDoC.

Among these three two‐target studies, one applied tDCS on the bilateral prefrontal cortex and was a study protocol with no result reported; one was about bilateral parietal regions to treat one MCS patient, demonstrating improvements in clinical scores and EEG and fMRI findings; and one chose bilateral M1 as the target, showing significant improvement in CRS‐R scores and greater alpha bandwidth in the parietal areas.

As for multi‐target therapy, one study applied tDCS in the prefrontal, left fronto‐temporo‐parietal cortex (FTPC), right FTPC, and left DLPFC; the results showed the tDCS group had a significant improvement in CRS‐R score and could increase the cortical connections both in the affected hemisphere and unaffected hemisphere. However, in another study of multi‐target therapy that applied tDCS in the frontoparietal areas (F3‐F4 and CP5‐CP6), a significant increase was noted in low‐frequency bands of EEG complexity, but on significant changes in behavioral examination in group level. The patients in this study received one 20‐min session of the therapy, which was not enough for pDoC.

Regardless of the format of tDCS or the selection of parameters, the research on tDCS made good progress in the last 5 years. The emergence of HD‐tDCS made up for the poor positioning accuracy of conventional tDCS. Home‐based tDCS made it possible to achieve the validity and be economical at the same time. Lots of trials have been conducted to confirm the effects of tDCS, and the outcomes could not draw a uniform conclusion for the heterogeneity of the protocols. Brain damage, electrode placement, and the number of sessions varied from study to study. Owing to the serious brain damage of pDoC, long sessions and multi‐target of tDCS might be more beneficial to patients. Moreover, MCS might be the main beneficiary seen from the above results. In addition, using biochemical testing techniques and neuroimaging and electrophysiological techniques as biomarkers to screen for tDCS responders is a trend in the current study. This may help clinicians to make new treatment options for patients whose behavioral assessment is UWS but mismatched with the neuroimaging and electrophysiological techniques. And at present, most of the studies are more about single‐center design and small sample sizes. There is still an urgent need to conduct multi‐center, large sample size studies to confirm the ideal protocol of tDCS for pDoC.

## 3 INNOVATIONS IN TRANSCRANIAL MAGNETIC STIMULATION

Transcranial magnetic stimulation (TMS) is a noninvasive intervention to arouse consciousness in pDoC. During the stimulation process, a specific coil is usually placed over the scalp to induce a magnetic field which will then be transformed into the intracranial currents to affect brain function.^33^ Though not recommended by the American Academy of Neurology in the practice guideline update of disorders of consciousness in the 2018 version,^34^ it is still a promising method to treat pDoC patients for the lack of effective therapies and the high cost of inpatient care. The characteristics of research on TMS published in the last 5 years are seen in Table 2.

**TABLE 2:** Studies of TMS published in the last 5 years.

Literature	Study design	Number of patients and Diagnosis	Frequency (Hz)	Target	Total number of pulses	Number of real rTMS sessions	Evaluations	Behavioral Outcomes	TMS induced effects
Liu et al. 2018^48^	Randomized, sham‐controlled study	2 UWS; 5 MCS	20	Left M1	1000	5	CRS‐R/resting state fMRI (functional connection)	Group level (−)	fMRI: Functional connection (+)
He et al. 2018^47^	Randomized, sham‐controlled, crossover study	3 UWS; 2 MCS; 1 eMCS	20	Left M1	1000	5	CRS‐R/resting state EEG (power spectra)	Group level (−)	EEG: In one patient: Four band power (+)
Xia et al. 2019^41^	Nonrandom controlled study	14 UWS; 7 MCS	10	Left DLPFC	1000	1	CRS‐R/TMS—EEG (TEP/TMS‐evoked connectivity)	MCS group (+)	TMS‐EEG: In MCS group: TMS‐evoked connectivity (+)
Legostaeva et al. 2019^46^	Nonrandom controlled study	16 UWS; 22 MCS	20	Left angular	3200	10	CRS‐R	MCS group (+)	—
He et al. 2020^45^	Nonrandom controlled study	25 UWS	20	Left DLPFC	2000	20	CRS‐R/resting state EEG	10/25 patients (+)	EEG: In responders: Alpha power (+) Delta power (−)
He et al. 2021^40^	Sham‐controlled study	25 UWS; 25 MCS	10 Hz	Left DLPFC	1000	10	CRS‐R/ estradiol levels	Group level (+)	Serum estradiol levels (+)
Ge et al. 2021^39^	Sham‐controlled study	32 UWS	10	Right DLPFC	1575	20	CRS‐R/MEP/CMCT	Group level (+)	MEP (−) CMCT (−)
Fan et al. 2022^49^	Randomized, double‐blinded, sham‐controlled trial	40 UWS	20	Left DLPFC	2000	20	CRS‐R	Group level (+)	—
Chen et al. 2022^44^	Randomized sham‐controlled study	50 pDoC	10	Left DLPFC	1000	30	CRS‐R/BAEP/SEP/GCS	Group level (+)	BAEP/SEP: N20‐P25 amplitudes (+), BAEP grade (+) N20 latencies (+)

Abbreviations: BAEP, brainstem auditory‐evoked potentials; CMCT, central motor conduction time; CRS‐R, JFK Coma Recovery Scale‐Revised; DLPFC, the dorsolateral prefrontal cortex; EEG, electroencephalography; eMCS, emerged from minimally conscious state; fMRI, functional magnetic resonance imaging; GCS, Glasgow Coma Scale; M1, the primary motor cortex; MCS, minimally conscious state; MEP, motor‐evoked potential; pDoC, prolonged disorders of consciousness; SEP, somatosensory‐evoked potential; TEP, TMS‐evoked potential; TMS, transcranial magnetic stimulation; TMS‐EEG, transcranial magnetic stimulation combined with electroencephalography; UWS, unresponsive wakefulness state.

### 3.1 Form of the stimulation

The forms of TMS are as follows: single‐TMS, paired‐pulse TMS, and repetitive TMS (rTMS).

Few studies have reported the use of single‐TMS in the protocol of pDoC. In general, it was used to determine the resting motor threshold (RMT). The RMT refers to the lowest intensity that could evoke the motor‐evoked potentials (MEPs) of which the amplitude is >50 mV peak‐to‐peak in at least five of 10 of the contractions by applying a figure‐of‐eight coil or a round coil over the motor cortex to deliver the single‐TMS.^35^ Although not being demonstrated in the therapy of TMS, two case reports had reported syncope by using single TMS in healthy subjects.^36^, ^37^ This may be a limitation of its use.

Paired‐pulse TMS could help show the signs of preserved brain connectivity in pDoC patients with noncommunicative brain damage but does not apply to the therapy of pDoC.

However, the most common form of the therapy used in TMS was repetitive. The rTMS refers to a mode that the coil generates a train of multiple pulses at a particular frequency. Under this stimulation mode, it is determined that low‐frequency (<1 Hz) has inhibitory effects that could reduce neuronal excitability, local metabolism levels, and cerebral blood flow, while high‐frequency (5–20 Hz) can increase cortical excitability. Moreover, it was noted that rTMS had prolonged effects and showed more efficacy in the therapy than other stimulation forms. In previous studies, only one reported the use of 5 Hz for the treatment of pDoC.^38^ Most of them were about 10 or 20 Hz. In the past 5 years, there were four articles about 10 Hz^39^, ^40^, ^41^, ^42^, ^43^, ^44^ and five articles about 20 Hz.^45^, ^46^, ^47^, ^48^, ^49^

### 3.2 Choice of target

As mentioned above, the recovery process of DoC is more relevant to the anterior forebrain mesocircuit and the frontoparietal network. The frontoparietal network contains two networks^50^, ^51^—the internal and external awareness network. The internal awareness network is more relevant to self‐awareness with respect to midline regions such as anterior cingulate cortex, precuneus/posterior cingulate cortex, and temporo‐parietal junctions and the hippocampi, while the external awareness network is relevant to the external environment mainly encompassing the lateral frontoparietal hemispheric regions. Similar to the choice of target in the protocol of tDCS, bilateral DLPFC and left M1 were also the most common targets in the TMS protocol. Six papers^39^, ^40^, ^41^, ^44^, ^45^, ^49^ reported its use in DLPFC, one session in left DLPFC^41^ showed positive results only in the MCS group, and the other five (four in left DLPFC,^40^, ^44^, ^45^, ^49^ one in right DLPFC^39^) all showed positive results in the real rTMS group. Two randomized sham‐control trials selected M1 as the target.^47^, ^48^ One of them invested in the functional connectivity effects of rTMS on pDoC.^47^ The other^48^ applied fMRI to analyze the functional connectivity, and it presented an enhanced trend after five sessions of rTMS in the left M1. However, these two did not show any positive results in group level in the behavioral assessment. In view of the inconsistency of results of most previous rTMS studies, some scholars suggested that DLPFC and M1 may not be the most ideal target.^48^ Thus, some researchers made efforts to seek new targets. Legostaeva et al.^46^ conducted a study applying rTMS in the angular gyrus. From the neuroanatomical position, the angular gyrus is a multimodal convergence hub with the default mode network (DMN); furthermore, it also connects with the frontoparietal control network. However, the outcomes showed effects on the MCS group, not the UWS group.

### 3.3 The methods of assessment on TMS

Most of the past studies have used the CRS‐R scale as well as electrophysiological methods such as EEG, TMS‐EEG, brainstem auditory‐evoked potential (BAEP), and somatosensory‐evoked potential (SEP) to assess the effectiveness and changes in brain activity evoked by TMS.^39^, ^41^, ^44^, ^45^, ^47^, ^49^ In the last 5 years, electrophysiological methods have remained the primary means of assessment. One study reported an increased central nervous system (CNS) conduction velocity and enhanced cortical activation of the superior reticular activation system after TMS, as evidenced by reduced MEP latency and faster central motor conduction time.^39^ Two papers reported the use of resting‐state EEG to assess brain function, both of which found that patients who showed progress may have had a high EEG power in the alpha band before TMS and a decrease in delta band after TMS.^45^, ^47^ Based on that, researchers suggested that the EEG power may be a biomarker for individualized therapy.^45^ One study applied TMS‐EEG to achieve the evaluation.^41^ TMS‐evoked potential (TEP) and global mean field amplitude (GMFP) were chosen to assess the reactivity of the brain while TMS‐evoked connectivity matrix was chosen to evaluate the effective connectivity between different regions of the brain. They found that the baseline of TEP and GMFA showed significant differences among healthy control, UWS, and MCS groups. This suggested that different levels of consciousness had different patterns of effective connectivity. MCS as well as the UWS groups did not observe any changes in TEP and GMFA after TMS. However, in terms of effective connectivity, enhanced connectivity was observed in healthy subjects and the MCS group, but not in the UWS group after TMS by analyzing the TMS‐evoked connectivity matrix.

In addition to electrophysiological techniques, imaging and neurophysiological techniques have also been used to evaluate the effects of TMS. Liu et al.^48^ used MRI to assess the resting state functional connectivity effects of rTMS. The result showed an enhanced trend in the left lateral parietal cortex, left inferior temporal cortex, and right DLPFC, but showed no significant changes in brain functional connectivity. In a study about neurophysiological technique,^40^ the researcher found that TMS could increase serum estradiol which is known to have a strong influence on cortical excitability. They suggested serum estradiol was a potential biomarker for TMS responders.

To date, the therapeutic effects of TMS remain inconsistent. Overall, all studies on TMS have common drawbacks. The sample size was small because of this, the reuse of patient data could not be avoided and the design of a randomized controlled trial was difficult to achieve. Moreover, there was great heterogeneity among patients such as different etiology, different age, different course of disease, and different lesion sites. As a consequence, the small sample size and large heterogeneity made it difficult to draw a unified conclusion about the effectiveness of TMS.

There is no denying that TMS is a promising treatment in pDoC, but the optimal parameters need further evaluation and validation. The current priority is to conduct multicenter, large‐sample, randomized controlled trials and establish a large sample database. Patient heterogeneity is a huge barrier in the progress of TMS. However, the rapid development of science brings the progress of technology and its renewal. The search for biomarkers in electrophysiological techniques, imaging techniques, and physiologically relevant techniques to screen TMS responders and develop individualized protocols is a major trend in current research.

## 4 TRANSCRANIAL ULTRASONIC STIMULATION

Transcranial ultrasonic stimulation (TUS) is a new form of neuromodulation. The energy delivered by TUS is a mechanical energy whose frequency is over 20,000 Hz. Ultrasonic stimulation has stronger penetration and higher spatial resolution than the two classical types of noninvasive neuromodulation (TMS and tDCS).^52^ Unlike TMS which only has an effect on the surface of the cortex, TUS can stimulate the regions 10–15 cm under the cranial bones.^52^ This feature makes it superior to other interventions to an extent, as it can stimulate deep brain regions like deep brain stimulation (DBS), but without any invasion. When it comes to classification, it can be divided into high (>200 W/cm^2^), medium (100–200 W/cm^2^), and low (<100 W/cm^2^) intensity according to the heat intensity.^53^ As for frequency, it also can be divided into focused (<1 MHz) and unfocused (1–15 MHz) ultrasound.^53^ Some animal experimental studies showed that low‐intensity TUS had neuromodulatory effects.^54^ Focused ultrasound has been considered to have neural regulation, but the current evidence shows that unfocused ultrasound may also achieve neuromodulation.^55^, ^56^

The vast majority of studies are about healthy humans. Among these studies,^55^, ^57^, ^58^, ^59^, ^60^, ^61^, ^62^, ^63^, ^64^ TUS has been proven to have a regulatory effect on the brain. However, few studies have explored the validity of TUS in treating pDoC. In 2016, TUS was first reported to be applied to arouse consciousness in DoC.^65^ Low‐intensity (720 mW/cm^2^), focused (650 kHz) TUS was used, directly aimed at the patient's thalamus. The protocol was 100 Hz repetitive pulse frequency with 0.5 ms pulse width, lasting 10 times. The CRS‐R score rose from 13 to 17 after the second session. Five days post‐stimulation, the diagnosis of the patient with TBI changed from MCS to emergence from a minimally conscious state (eMCS). The outcome was inspiring, but the duration of DoC in the patient was only 19 days, thus making it difficult to classify as pDoC.

In the last 5 years, just one case report revealed the use of TUS in the treatment of pDoC.^66^ Three pDoC patients with different etiologies were enrolled in this study, including hemorrhagic stroke, cardiac arrest/hypoxia, and motor vehicle accident/TBI. The stimulation parameters were almost the same as in the literature described above. The intensity was set as 719.73 mW/cm^2^, carrier wave frequency was 650 kHz, pulse width was 0.5 ms, pulse frequency was 1000 Hz, and the target was the thalamus. However, they only received two‐session stimulation. Two of the three patients responded immediately after receiving TUS treatment. The diagnosis of patient 1 changed from the minimally conscious state plus (MCS+) to eMCS, but it regressed to MCS+ after follow‐up visits. The baseline state of patient 2 was a minimally conscious state minus (MCS−), which changed to MCS+ after the first single session and was maintained at MCS+ throughout the follow‐up assessments. Patient 3 whose diagnosis was MCS+ did not show any change during the two‐session intervention but changed from MCS− to MCS+ in the first follow‐up visit.

Some researchers have probed into the restorative strategies applied in DoC.^67^, ^68^ All studies showed that the methods promoting the recovery of consciousness were in the form of directly and indirectly exciting the thalamus. DBS is a means of neuromodulation that can achieve precise stimulation of the thalamus and obtain remarkable results. However, the defects of DBS include its invasiveness, high cost, and ethical issues, which limit its application.^69^ Thus, an alternative means of treatment is important. TUS is a brand‐new intervention of neuromodulation in treating pDoC. Low‐intensity focused ultrasound has the features of less energy aberration and precise penetration. Furthermore, it is noninvasive and cost‐effective without any ethical issues and can replace DBS in terms of precise stimulation of the thalamus. In conclusion, TUS has a promising future in treating pDoC, but it still needs randomized controlled trials to further explore its efficacy and safety.

## 5 MEDIAN NERVE ELECTRICAL STIMULATION (MNS)

Consciousness is composed by two parts: arousal and awareness. Arousal is considered mostly related to the ascending reticular activating system (ARAS).^70^, ^71^ ARAS consists of neural circuits connecting the brainstem to the cortex. The median nerve is part of the peripheral nerve, and its spinal segments are associated with ARAS neurons.^72^ It is considered the peripheral gateway to the central nervous system because the area it innervates occupies a large area in the central nervous system. Consequently, stimulating the median nerve can realize the step‐by‐step activation of the median nerve—spinal nerve, followed by cervical spinal cord, brainstem, thalamus, and cortex—hence activating ARAS and promoting the recovery of consciousness.^72^

As early as the 1990s, MNS was in use to treat DoC patients. Cooper in 1999^73^ and Peri in 2001^74^ applied the right MNS to stimulate comatose patients. The outcomes showed that the Glasgow Coma Scale (GCS) scores improved and the days of hospitalization decreased. In 2003, Liu et al.^75^ utilized the right MNS to awaken consciousness of patients, which was followed by SPECT scanning after all stimulation protocols. The results demonstrated that the right MNS could help patients elevate the cerebral blood flow and regained consciousness. Furthermore, the efficacy is age‐related. Subsequently, large samples of controlled clinical trials^76^ were reported to validate its effectiveness.

As all the outcomes showed its effects on recovery of consciousness and no side effects were observed, it may be an easy, effective, and noninvasive technique in this field. However, all patients were related to a comatose state. More importantly, this technique did not show any improvement in the last 5 years after one multi‐center, prospective, randomized controlled trial was reported in 2017.^77^

## 6 TRANSCUTANEOUS AURICULAR VAGUS NERVE STIMULATION (taVNS)

Vagus nerve stimulation (VNS) has two forms, invasive and noninvasive. The auricular branch of the VN is the only branch that reaches the body surface.^78^ Thus, taVNS is a noninvasive method of VN. Auricle stimulation has a long history of development that can be traced back to 500 BC.^79^, ^80^ Then, taVNS was gradually used to treat various diseases both in Eastern and Western civilizations.

The exact mechanism underlying taVNS is still unknown. From the anatomical perspective, the afferents of the auricular vagus nerve first enter the jugular ganglion, then the vagus trunk, and finally reach the nucleus tractus solitarius. When information from the afferent trunk is collected, the caudal ventrolateral medulla and dorsal motor nucleus get activated, which then regulate the central autonomic activity.^81^, ^82^ Recently, one study proposed a vagal cortical pathways model. Based on that, six different mechanisms of taVNS were proposed for consciousness recovery.^83^

The taVNS is used to more applied to epilepsy and depression. Until 2017, there was only one case report^84^ which showed the use of taVNS in pDoC. Briefly, taVNS was used in a 73‐year‐old female who was in VS. After 4 weeks of stimulation (target: bilateral ear concha; frequency: 20 Hz; intensity: 4–6 mA; duration: twice daily for 30 min), her CRS‐R score increased from 6 to 13, and the motor and oromotor functions showed new behaviors consistent with the diagnosis of MCS. The fMRI showed increasing functional connectivity between posterior cingulate/precuneus and hypothalamus, thalamus, ventral medial prefrontal cortex, and superior temporal gyrus and decreasing functional connectivity between the posterior cingulate/precuneus and cerebellum. Noé et al. conducted a prospective study in 2020^85^ to investigate the effectiveness of taVNS in pDoC. Fourteen patients (six VS/UWS and eight MCS) were enrolled in this study. The protocol was set as follows: target: left ear concha; frequency: 20 Hz; intensity: 1.5 mA; duration: twice daily for 30 min, 4 weeks (5 days/week). After a 4‐week stimulation, VS patients did not observe any improvement in the CRS‐R score, while only one of the eight MCS patients improved. However, another four MCS patients showed different degrees of improvement at the 1‐month follow‐up. No severe side effects were reported in the above two trials.

Although the effectiveness of taVNS has been preliminarily verified, the sample capacity of the current trial is small and the parameters are not unified. Thus, large samples of clinical trials, especially randomized controlled trials are needed for further verification.

## 7 CONCLUSION

The improvement of emergency techniques has improved the survival rate of patients with severe brain injury and has also led to a massive rise in the number of pDoC from severe craniocerebral injuries. However, the search for interventions backed by evidenced‐based medicine is currently a challenge for regaining consciousness in pDoC. Noninvasive neuromodulation is a promising approach in this field, given that it is noninvasive, with few side effects, and does not involve ethical issues. It contains many types such as tDCS, TMS, TUS, taVNS, and MNS. Though all these belong to noninvasive neuromodulations with the above advantages, they still have many differences (Table 3). From the perspective of stimulus, tDCS, taVNS, and MNS use the current, TMS applies impulse magnetic field and TUS uses low‐intensity focused ultrasound. For site of action, tDCS and TMS mainly act on the cerebral cortex, TUS mainly on the cortex and deep subcortical area, and taVNS and MNS mainly on peripheral nerves. In terms of progress achieved, tDCS has made the most. The conventional medical mode transformed to the home‐based mode, which enabled translation from clinical to domestic use, achieving portability and broad utility. At the same time, the big pad electrodes with rough stimulation positioning and easy current dissipation were replaced by a high‐definition electrode with precise stimulation. Besides, in order to activate a greater range of cortex and better improve the levels of consciousness, the selection of the choice of tDCS targets has changed from single one to two‐targets or even multi‐targets. And the session has also transitioned from single mode to multiple mode. TMS has also made some progress. Its target selection shifted from the less effective M1 to the DLPEC and then one parietal cortex (angular gyrus). Because they have been used for a long time to promote the recovery of consciousness in pDoC, tDCS, and TMS are the closest to being formally used in clinical practice. TUS is a relatively new intervention in pDoC, the great advantage of it is the ability to activate the central thalamus which is closely related to consciousness. However, due to its short application time on pDoC and the fact that the related studies are more about case reports, a formal treatment protocol has not yet been established. MNS is a peripheral stimulation that acts on the median nerve. The research mostly focused on coma patients. Only a few domestic studies investigated its effects in pDoC, but the effects need further validation. Also serves as a peripheral stimulation, the taVNS's application on pDoC experienced not a very long time. Thus, its safety and efficacy also need further studies. For etiology, researchers found that the prognosis of traumatic pDoC was better than nontraumatic pDoC. However, due to difficulties in patient recruitment and some clinical limitations, subgroup analyses based on etiology are hard to achieve. Though more research is expanding the sample size, it is hard to draw a conclusion that one patient with a certain etiology is exactly suitable for a particular noninvasive neuromodulation.

**TABLE 3:** The comparison of the neuromodulations in pDoC.

Intervention	Stimulus	Action site	Indications	Contraindications	Adverse reaction	Merits	Demetris
tDCS	Current	Cortex	VS/UWS MCS Stable disease	Pacemaker implantation Intracranial mental implants Massive cerebral infarction Acute cerebral hemorrhage Intracranial hypertension Lesion or inflammation in target sites Hemorrhagic tendency	Tingling sensation Itching Dizzy Headache Burn	Portability Easy operation Less cost High safety	Low current density Low spatial resolution Low depth
TMS	Impulse magnetic field	Cortex	VS/UWS MCS Stable disease	Epilepsy Pacemaker implantation Intracranial mental implants Cochlear implants Cardiac stent Intracranial hypertension Lesion or inflammation in target sites	Syncope Epilepsy Tingling sensation Itching Dizzy Headache	Precision Easy operation High safety	Low depth Demanding positioning
TUS	Ultrasound	Cortex Deep cerebrum (Central thalamus)	VS/UWS MCS Stable disease	Organ dysfunction Hemorrhagic tendency Acute infectious diseases Cochlear implants Skin damage in target sites	Dizzy Tissue injury	High spatial resolution Precision Deep stimulation Compatibility	Thermogenesis Few clinical application
MNS	Current	Peripheral nerve (Median nerve)	Coma	Frequent arrhythmias Frequent epilepsy Cochlear implants Organ dysfunction Gravida	Pain	Portability Easy operation Less cost High safety Early intervention	Main used in coma Few clinical application
taVNS	Current	Peripheral nerve (Vagus nerve)	VS/UWS MCS Stable disease	Severe diseases such as malignant tumors Incomplete vagus nerve Severe arrhythmia Heart rate below 60 Cochlear implants	Changes in voice Cough Sore throat Dyspnea Paraesthesia Dysphagia Headache	Portability Easy operation Less cost High safety	Affecting respiratory Few clinical application

Abbreviations: MCS, minimally conscious state; MNS, median nerve electrical stimulation; taVNS, transcutaneous auricular vagus nerve stimulation; tDCS, transcranial direct current stimulation; TMS, transcranial magnetic stimulation; TUS, transcranial ultrasonic stimulation; VS/UWS, vegetative state/unresponsive wakefulness syndrome.

In summary, the noninvasive neuromodulations mentioned above have made varying degrees of progress in promoting the recovery of consciousness in pDoC. Though numerous studies have shown their progress in the last 5 years, the results also showed that they were nonuniform, the sample size was small, the heterogeneity of patients was high, and the stimulus parameters were inconsistent. More randomized controlled studies are needed to confirm the ideal parameters and provide evidence‐based medical support for noninvasive treatment in pDoC.

## AUTHOR CONTRIBUTIONS

**Xiaoping Wan:** Writing – original draft; data curation. **Ye Zhang:** Writing – review and editing; funding acquisition. **Yanhua Li:** Data curation. **Weiqun Song:** Funding acquisition; conceptualization.

## FUNDING INFORMATION

This work was supported by STI2030‐Major Projects (grant numbers: 2021ZD0204300, 2021ZD0204305).

## CONFLICT OF INTEREST STATEMENT

The authors declare no conflicts of interest.

## Data Availability

The data that support the findings of this study are available on request from the corresponding author. The data are not publicly available due to privacy or ethical restrictions.
